# The Effect of Neuroepo on Cognition in Parkinson’s Disease Patients Is Mediated by Electroencephalogram Source Activity

**DOI:** 10.3389/fnins.2022.841428

**Published:** 2022-06-30

**Authors:** Maria L. Bringas Vega, Ivonne Pedroso Ibáñez, Fuleah A. Razzaq, Min Zhang, Lilia Morales Chacón, Peng Ren, Lidice Galan Garcia, Peng Gan, Trinidad Virues Alba, Carlos Lopez Naranjo, Marjan Jahanshahi, Jorge Bosch-Bayard, Pedro A. Valdes-Sosa

**Affiliations:** ^1^Ministry of Education (MOE) Key Lab for Neuroinformation, The Clinical Hospital of Chengdu Brain Science Institute, University of Electronic Science and Technology of China, Chengdu, China; ^2^International Center of Neurological Restoration (CIREN), La Habana, Cuba; ^3^Cuban Neuroscience Center (CNEURO), La Habana, Cuba; ^4^UCL Queen Square Institute of Neurology, London, United Kingdom; ^5^McGill Centre for Integrative Neuroscience, Montreal Neurological Institute, Montreal, QC, Canada

**Keywords:** Neuroepo, EEG, Parkinson’s disease, source analysis, Canonical Correlation Analysis (CCA), whitening

## Abstract

We report on the quantitative electroencephalogram (qEEG) and cognitive effects of Neuroepo in Parkinson’s disease (PD) from a double-blind safety trial (https://clinicaltrials.gov/, number NCT04110678). Neuroepo is a new erythropoietin (EPO) formulation with a low sialic acid content with satisfactory results in animal models and tolerance in healthy participants and PD patients. In this study, 26 PD patients were assigned randomly to Neuroepo (*n* = 15) or placebo (*n* = 11) groups to test the tolerance of the drug. Outcome variables were neuropsychological tests and resting-state source qEEG at baseline and 6 months after administering the drug. Probabilistic Canonical Correlation Analysis was used to extract latent variables for the cognitive and for qEEG variables that shared a common source of variance. We obtained canonical variates for Cognition and qEEG with a correlation of 0.97. Linear Mixed Model analysis showed significant positive dependence of the canonical variate cognition on the dose and the confounder educational level (*p* = 0.003 and *p* = 0.02, respectively). Additionally, in the mediation equation, we found a positive dependence of Cognition with qEEG for (*p* = < 0.0001) and with dose (*p* = 0.006). Despite the small sample, both tests were powered over 89%. A combined mediation model showed that 66% of the total effect of the cognitive improvement was mediated by qEEG (*p* = 0.0001), with the remaining direct effect between dose and Cognition (*p* = 0.002), due to other causes. These results suggest that Neuroepo has a positive influence on Cognition in PD patients and that a large portion of this effect is mediated by brain mechanisms reflected in qEEG.

## Introduction

Parkinson’s disease (PD) is one of the most common neurodegenerative disorders. The global burden of PD has doubled in 26 years – from 2 million patients in 1990 to 6 million patients in 2016 (Feigin et al., 2017). The clinical symptoms of PD include motor symptoms such as tremor, slowed movement, muscle rigidity, and impaired balance and gait. But it is essential to underline that in addition to motor symptoms may also manifest as cognitive impairment, depression, anxiety, apathy, hallucinations, and sleep disorders. These symptoms affect daily life activities, with a strong negative impact on quality of life (Schrag et al., 2000; Rahman et al., 2008) and their caregivers (Schrag et al., 2006). With the management of such cognitive consequences of PD, we are mainly concerned here. For a review see Aarsland et al. (2021).

Although levodopa and dopamine agonists initially control and improve the motor symptoms of PD patients, there is still no effective intervention to slow down the progression of the illness, including cognitive deterioration. A contribution to the solution of PD disease burden might be neuroprotective drug therapy. However, despite keen interest in this approach, the issue of neuroprotection has proved to be complex because no fully demonstrated therapeutic agents are yet available (Athauda and Foltynie, 2015). Contributing to this lack of results is a lack of validated biomarkers. This article addresses these issues in the framework of a safety trial for a new neuroprotective molecule, erythropoietin (EPO).

Erythropoietin was initially discovered as a hematopoietic growth factor. However, it has proved to be a promising molecule for treating neurological diseases (McPherson and Juul, 2008; Merelli et al., 2015). There is increasing evidence that this molecule plays a significant role in neural survival and functional recovery in animal PD models (Rey et al., 2019). Neuroprotection has been confirmed even with different administration strategies: intraventricular, intrastriatal, gene therapy, or grafted dopamine survival (Kanaan et al., 2006; Xue et al., 2007, 2010; Kadota et al., 2009).

In clinical models, EPO has also been tested with reliable results. Pedroso et al. (2012) conducted a study on (*n* = 10) PD patients to evaluate the neuroprotective effect of Cuban recombinant human erythropoietin (ior-EPOCIM). They found that the drug was safe and well-tolerated (Pedroso et al., 2012). PD patients treated had clinically positive and statistically significant cognitive changes after the treatment. Jang et al. (2014) also confirmed that recombinant human erythropoietin (rhEPO) was safe and had beneficial effects on non-motor symptoms (Cognition, mood, and sleep/fatigue) of PD patients (Jang et al., 2014). However, rhEPO requires high doses and prolonged application, with the danger of producing adverse effects because of hematocrit and blood viscosity increment.

For this reason, a new formulation of EPO with a low content of sialic acid has been developed, known as Neuroepo. This molecule is similar to that produced in the brain of mammals, maintaining its neuroprotective properties as shown in *in vitro* models (Garzón et al., 2018b) and animal experiments (Rodríguez Cruz et al., 2010, 2016). These studies showed that Neuroepo has the required lack of inducer effect on the synthesis of erythrocytes. Furthermore, Neuroepo was shown to be safe and well-tolerated in healthy people (Santos-Morales et al., 2017) and PD patients (Garcia Llano et al., 2021).

Beyond safety, our group wished to assess the possible cognitive effects of Neuroepo on PD patients. We reported preliminary results on cognitive performance (Pedroso et al., 2018). That study evaluated univariate longitudinal differences after 6 months of treatment (or placebo), yielding a positive effect of the drug on some cognitive variables. In this article, we carry out a more thorough analysis of the data initially reported, analyzing whether the resting state electroencephalogram (EEG), as a direct reflection of brain activity, does indeed mediate the changes produced by Neuroepo in cognitive performance in these patients.

Electrophysiology has long been proposed as a reliable biomarker to discriminate between PD patients with healthy controls (Waninger et al., 2020) and to evaluate the progression of PD patients and their cognitive impairment and decline, as reviewed in Cozac et al. (2016) and Shirahige et al. (2020) for quantitative EEG (qEEG) and Seer et al. (2016) for ERPs. Additionally, recent studies have shown an association between qEEG parameters and cognitive variables during disease progression in PD (Caviness et al., 2007; Klassen et al., 2011; Gu et al., 2014; Babiloni et al., 2017). Indeed, several longitudinal PD studies go beyond the mere correlation of qEEG and cognitive descriptors. They show that qEEG predicts future cognitive deterioration and neurodegeneration (Klassen et al., 2011; Arnaldi et al., 2017; Caviness et al., 2018; Betrouni et al., 2019). There is thus encouragement in the literature to use both purely cognitive and qEEG variables to evaluate the effect of neuroprotective drugs on PD cognitive progression.

Nevertheless, the studies cited evaluate cognitive or qEEG variables as completely separate modalities. Recent work in the multi-omics and multidomain search for brain biomarkers has stressed, to great advantage, extracting underlying latent variables to focus on shared sources of variability (Smith and Nichols, 2018; Liu et al., 2021; Zhang et al., 2022). Many of these proposals focus on modern variants of Canonical Correlation Analysis (CCA), a significant application to PD described in Cai et al. (2022). This approach is the one we follow in this article identifying latent variables for Cognition and qEEG. Furthermore, we leverage recent advances in causal counterfactual mediation analysis (Imai et al., 2010) to assess our PD clinical trial data. Specifically, we test (a) whether Neuroepo affects the latent cognitive variable; (b) whether the qEEG latent variable mediates any such effect.

## Materials and Methods

### Sample Description

The study was a physician-led safety trial with a double-blind design conducted at the International Neurological Restoration Center (CIREN in Spanish), Cuba, collaborating with the Center for Molecular Immunology (CIM in Spanish). The study was registered at clinicaltrials.gov/ct2/show/NCT04110678?term=neuroEPO&cond=Parkinson&cntry=CU& draw=2&rank=1 with the number NCT04110678.

Participants were recruited in the outpatient Movement Disorders Clinic at CIREN from February 2015 to July 2016. Twenty-six patients with a clinical diagnosis of PD were selected from 46. The inclusion criteria for the selected sample met the criteria of the UK Brain Bank, severity stages of Hoehn and Yahr (1967) I–II, age between 40 and 70, good response to dopaminergic medication: 30% of change of motor symptoms as rated on the Movement Disorder Society-Unified Parkinson Disease Rating Scale (Goetz et al., 2007), good general health, without depression or cognitive impairment. The exclusion criteria were stringent and eliminated patients with cognitive deterioration, psychiatric disorders, pregnant women, hypertension or any non-compensated diseases, sepsis, or treatment with other drugs. The detailed list of inclusion criteria can be found in Garcia Llano et al. (2021). The severity of the PD was evaluated by the Hoehn and Yahr scale.

The 26 patients were randomly divided into two groups: One group consisted of 15 patients who received a 1 mL dose of intranasal Neuroepo per week for 5 weeks. Another group included 11 patients who took a placebo.

### Patient Assessment

The evaluation of the motor function was performed by a neurologist of the Movement Disorders Clinic at CIREN, using the Movement Disorder Society motor scale (Unified Parkinson’s Disease Rating Scale section motor; UPDRS III).

We applied the levodopa equivalent dose (LED) method to standardize the intake of pharmaceutical agents by the patients. The LED was calculated using the conversion proposed by Schade et al. (2020). See Table 1 for the demographic and clinical description of this sample.

**TABLE 1:** Clinical and demographic characteristics of the sample.

Baseline assessment		Neuroepo	Placebo	Total	*P*-value
Total		*N* = 15	*N* = 11	*N* = 26	
Age	Years (mean, SD)	56.4 ± 7.8	60.9 ± 7.2	58.4 ± 7.6	*p* = 0.16
Gender	Male	7 (46.6%)	8 (72.7%)	15 (55%)	*p* = 0.18
	Female	8 (53.4%)	3 (27.2%)	11 (45%)	
Hoehn and Yahr (severity)	I	4 (26.6%)	1 (9.09)	5 (19.2%)	*p* = 0.26
	II	11 (73.4%)	10 (91%)	21 (80.8%)	
Duration of illness	Years (mean, SD)	5.4 ± 3.2	5.8 ± 4.1	5.6 ± 3.5	*p* = 0.98
PD familial antecedents	Yes	6 (40%)	3 (27.2%)	9 (34.6%)	*p* = 0.5
	No	9 (60%)	8 (72.8%)	17 (65.4%)	
Levodopa equivalent dose (LED)	Daily dose (mean, SD)	935.83 ± 302.54	939.3 ± 194.0		*p* = 0.77

The EEG, neuropsychological, and motor evaluations were performed at two points: at the baseline and 6 months later. The patients were studied in the “ON” state for all the assessments planned during the early mornings. The patients were advised to take breakfast and their regular medication before attending the evaluations.

Other assessments included clinical blood tests and blood pressure to follow the health status of the patients (data not shown).

The project had ethics approval from the institutional review board at CIREN. Written informed consent was obtained from all patients and caregivers following the ethics standards at CIREN and CIM.

### Neuropsychological Assessment

Cognitive performance was assessed with a comprehensive neuropsychological battery for global cognitive screening, comprising 32 variables from 9 tests. The tests included the Mini-Mental State Examination (Folstein et al., 1983) and the Dementia Rating Scale (Mattis, 1988); the Rey Auditory Verbal Learning test (Bean, 2011), the subtest letter-number sequencing of the Working Memory Index of WAIS III (Wechsler, 1997), the Rey Complex Figure, copy and delayed recall (Rey, 1941), Delis-Kaplan verbal fluency (Delis et al., 2001), Trail-Making (Reynolds, 2002) the Stroop color-word Interference test (Rivera et al., 2015), and the Frontal Assessment Battery (Dubois et al., 2000).

The initial 32 variables were screened to leave only those with a significant difference in *t*-tests between the two measurement points for the Neuroepo and control groups. FDR set the threshold with both *p* and *q* levels set to 0.05. Consequently, the final six neuropsychological variables included are described in Table 2.

**TABLE 2:** Cognitive tests items used.

Abbreviation	Specific test and maximum value
DRS	DRS Total (max = 144)
MMSE	Mini Mental State Examination (max = 30)
FAB	Frontal Assessment Battery. Total (max = 18)
Sequency	Working Memory Index WAIS III. Subtest: letter-number sequencing (max = 16)
Memory	Rey Figure Recall (max = 36)
Recognition	Rey Auditory Verbal Learning Test (RAVLT). Subtest: Recognition (max = 15)

The mood of the patients was assessed by the Hospital Anxiety and Depression Scale (HADS) validated for the Spanish population (Herrero et al., 2003) only at the baseline to decide the final inclusion of the patients.

### Electroencephalogram Recording and Preprocessing

Eyes closed resting monopolar EEG was recorded from Fp1, Fp2, F3, F4, C3, C4, P3, P4, O1, O2, F7, F8, T3, T4, T5, T6, Fz, Cz, and Pz using the International 10/20 system, all referenced to the linked earlobes [A1 (left)-A2 (right)]. The EEG was recorded using a Cuban neurometric system Medicid IV system and the Track Walker TM v5.0 software^1^ at the CIREN Neurophysiology Lab. For all electrodes, the impedance did not exceed 5 kΩ. EEG signals were amplified 10,600-fold, bandpass filtered from 0.5 to 30 Hz, and sampled by a 12-bit analog-to-digital converter at 200 Hz.

The EEG was recorded at two assessment points: at baseline before the intervention and 6 months after administering the drug or placebo. We selected 2.56-s artifact-free segments selected by an expert (TVA) for the analysis using visual inspection. For the removal of other artifacts due to interfering physical or physiological activities, we employed different semi-automatic plugins based on EEGLAB (Delorme and Makeig, 2004) under the Matlab platform. Because of artifacts and poor quality EEG recordings, one participant from the placebo group was eliminated from the final sample. Thus, the final sample for the analysis was Neuroepo *n* = 15, placebo = 10, total *n* = 25, reduced from the original *n* = 26.

### Quantitative Electroencephalogram Analysis

The topographic (qEEG) and tomographic (qEEGt) quantitative EEG methods have been explained elsewhere (Taboada-Crispi et al., 2018; Bringas Vega et al., 2019; Bosch-Bayard et al., 2020) and are now implemented as the qEEGt plugin at the CBRAIN (Bosch-Bayard et al., 2020) platform, available from the CBRAIN portal: https://portal.cbrain.mcgill.ca.

For the Quantitative EEG analysis (qEEG), the EEG signals in time were re-reference to the Average Reference montage and corrected by the Global Scale Factor (Hernandez et al., 1994). This factor accounts for variability in the EEG due to factors unrelated to the neurophysiology, such as the skull thickness, hair volume, impedance, recording conditions, and others, which may introduce a baseline bias affecting the spectra from different participants. The EEG was then transformed to the frequency domain using the Fast Fourier Transform (FFT). An average of 24 epochs of EEG artifact-free and quasi-stationary signals, manually selected by the expert neurophysiologists, were used to calculate the Power Spectral Density (PSD), both at the scalp and well at the sources. Each epoch comprised 512-time samples (i.e., 2.56 s). The transformation to the frequency domain was carried out using Bartlett’s method (Møller, 1986) by averaging the cross-periodograms. This procedure yielded 49 cross-spectral matrices for the 19 electrodes in a frequency range from 0.78 to 19.14 Hz, with frequency bins every 0.39 Hz.

For the calculation of the PSD at the EEG sources, the VARETA (Variable Resolution Electromagnetic Tomography) methodology, as implemented in the Neuronic software (Bosch-Bayard et al., 2001) and CBRAIN plugin (Bosch-Bayard et al., 2020) was used. VARETA is a discrete spline EEG inverse solution based on a forward model that incorporates anatomical constraints using the template of ICBM (probabilistic brain atlas) created by the Montreal Neurological Institute (MNI) (Evans et al., 1993). Source spectra were then Log-transformed to achieve an approximately Gaussian distribution.

### Statistical Analysis

To recap, the data analyzed comprised these measures:

• The confounding variables for each patient were education, severity (Hoehn & Yahr scale), and age when entering the trial.
• The primary variables were measured simultaneously on two occasions (baseline and 6 months later) regarding their entry into the trial. These are considered repeated measures:
  a. Dose: the amount of Neuroepo received by each patient. At the baseline, all the patients had dose = 0. Six months later, only the Neuroepo group had received 5 mg. We do not use the group as a main or fixed effect in our analysis.
  b. Quantitative EEG variables for each subject: 3,244 log source spectra measured at 49 frequency points.
  c. Cognitive variables for each subject: MMSE, DRS, FAB, Sequency, Memory, and Recognition.

To deal with the high dimensionality of data and small sample size, we employed probabilistic Canonical Correlation Analysis (pCCA) to obtain cognitive and qEEG cognitive latent variables reflecting common sources of variation. We then implemented a simple causal (counterfactual) mediation model with repeated measures to investigate the dose-effect on qEEG and cognitive variables using these latent variables.

### Data Whitening/Sphering/Probabilistic Canonical Correlation Analysis

Whitening, or sphering, is a preprocessing step for data analysis and machine learning models. A set of random variables with a known covariance matrix is transformed into a latent space with identity covariance (Zhang, 1998). Whitening is used to achieve unique features in data and remove redundant information. It also fulfills the purpose of data compression (Kessy et al., 2018). Whitening can be formally expressed as:


(1) $$Z=W_{z}\,Z:\Sigma_{Z}=I$$

*Z* is the observed variable, *Z* is the latent whitened variable, and *Wz* is the whitening matrix or unmixing matrix. A recent study implemented pCCA as a whitening transform to integrate high-dimensional gene expression and methylation data for lung carcinoma (Jendoubi and Strimmer, 2019). The purpose of using CCA for constructing *Wz* is twofold. It simultaneously whitens two variables while constraining the cross-correlation matrix to achieve highly correlated unique features between two measured variables. Jendoubi and Strimmer (2019) also demonstrated that their implementation of CCA can identify both positive and negative associations (unlike classic CCA, which allows only positive correlations).

We have implemented data whitening with pCCA to integrate qEEG and Cognitive variables using the whitening package in R (Strimmer et al., 2021). The first factor from whitened qEEG and Cognition (*qEEG*,*Cog*) was used for the mediation analysis.

### Mediation Model

**FIGURE 1:**
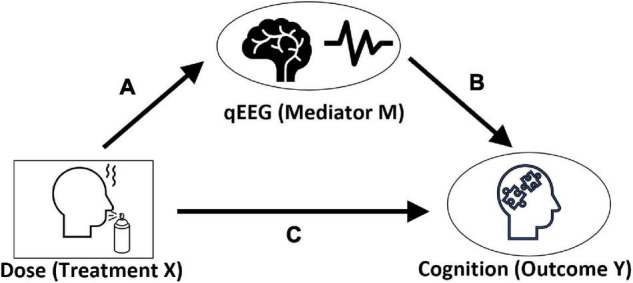
A mediation model. Effect of dosage on Cognition *via* qEEG. All three are repeated measures at two-time points. Ovals are latent variables, whereas rectangle shows observed variables. In this causal diagram, path c is the “direct effect of the Dose on Cognition.” Path following links a and b represent the “mediation effect” we wish to test.

To investigate if the effect of Neuroepo dosage on Cognition is mediated by qEEG, we implemented the mediation model shown in Figure 1.

In this model Dosage of Neuroepo is the treatment variable. The latent variables qEEG (*qEEG*) and Cognition (*Cog*) are the mediator and outcome, respectively. The significance of the paths is analyzed using the following general repeated-measures, mixed-effect models:


(2) Y=X⁢β+S⁢θ+ε

Here *Y* is the outcome variable, *X* and *S* are the fixed and random effects covariates. β and θ are estimated parameters for fixed and random effects. This general model was instantiated in two specific linear mixed effect models:

1. Mediator model: This model was implemented to estimate the dependence of qEEG on the Neuroepo dose (Figure 1: path a). We have included disease severity, subject’s education, and age as confounding factors

(3) q⁢E⁢E⁢G∼ 1+Dose+severity+education+a⁢g⁢e.t⁢r⁢i⁢a⁢l+random⁢(S⁢u⁢b⁢j⁢e⁢c⁢t⁢s)

2. Outcome model: This model was used to estimate the two paths (Figure 1: paths b and c) to the outcome:
  • The path between outcome and mediator (Figure 1: path b), which is from qEEG to Cognition
  • The direct path from treatment to outcome (Figure 1: path c) which is from Neuroepo dosage to Cognition

(4) C⁢o⁢g∼ 1+q⁢E⁢E⁢G+Dose+severity+education+⁢a⁢g⁢e.t⁢r⁢i⁢a⁢l⁢+random⁢(S⁢u⁢b⁢j⁢e⁢c⁢t⁢s)

These models were fitted using the lme4 package (Bates et al., 2015) from R. We also performed a power analysis using the “simr” package in R, which runs the simulation-based power analysis for linear mixed effect models (Green and MacLeod, 2016). Power analysis for both models was carried out with 100 simulations. A statistical power value threshold of 80% or more was chosen to identify adequate statistical power.

Furthermore, we combined the estimates of these models to compute the direct (Figure 1: path c) and indirect effects (joint estimate for Figure 1: paths a and b) between Neuroepo dosage and latent cognitive variable. Estimates for the direct and indirect path are calculated *via* the mediation package (Tingley et al., 2019) in R with 1000 Monte-Carlo simulations.

## Results

### Demographics

Table 1 shows the demographic and clinical characteristics of the sample.

The average age of patients in the Neuroepo group was 56.4 years old (SD = 7.8), and the average age for the placebo group was 60.9 years old (SD = 7.2). The average duration of the disease for patients in the two groups was 5.4 years (SD = 3.2) and 5.8 years (SD = 4.1), respectively. Using the Hoehn and Yahr scale, we classified four patients in the Neuroepo group and one in the placebo group at stage I. We also identified eleven patients from the Neuroepo group and ten in the placebo group at stage II. Only nine patients had familial antecedents of PD (six patients in the Neuroepo group and three in placebo). No significant differences were found between the two groups concerning age (*p* = 0.16), duration of illness (*p* = 0.98), familial antecedents of PD (*p* = 0.5), or severity of PD (*p* = 0.26.). Additionally, we found no significant differences between baseline and 6-months after intervention in both groups for the total score of the UPDRS “on” (*p* = 0.71) and “off” (*p* = 0.88), neither the LED (*p* = 0.77). There were no significant differences in mood between both groups at baseline.

### Extraction of Cognitive and Quantitative Electroencephalogram Latent Variables

The whole qEEG spectra of (3,244 voxels × 49 frequency points) for each subject were used to compute the pCCA whitening transform. For this analysis, the six cognitive scores selected: (DRS, FAB, MMSE, Sequency, Memory, and Recognition) were included in calculating the latent cognitive scores. pCCA whitening was carried out to find a joint transform for the cognitive scores and the complete set of qEEG variables.

The pCCA of these two datasets showed that the first latent dimension shows the strongest positive association between qEEG and Cognition. We restricted the further analysis to the first dimension, which was most highly associated with a canonical correlation of 0.97.

**FIGURE 2:**
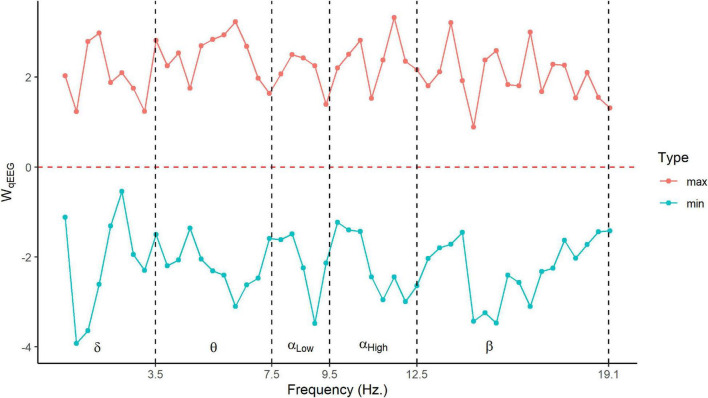
Frequency marginal distribution of qEEG loading. Minimum and maximum loadings of *W*_*qEEG*_for 3,244 sources at each frequency point. The *y*-axis is pCCA loadings for scaled data, and the *x*-axis is the frequency in Hz. Here we are using the classic frequency bands: delta (δ) = 1–4 Hz, theta (θ) = 4–8 Hz, alpha (α) = 8–12.5 Hz, and beta (β) = 12.5–20 Hz.

**FIGURE 3:**
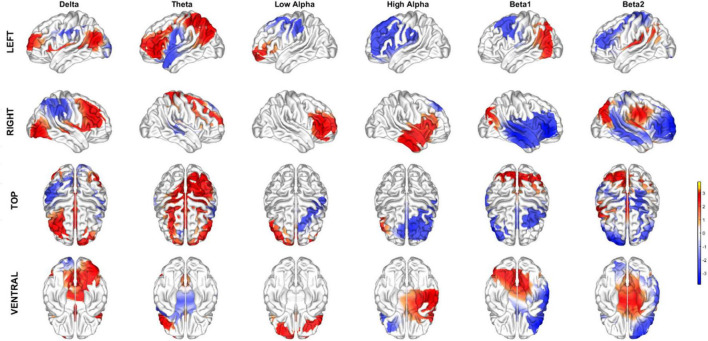
Topographic maps of the sources corresponding to the loadings of the qEEG latent variable for each classical frequency bands.

Table 3 and Figures 2, 3 show the loadings of the first dimension from CCA whitened qEEG and Cognition.

**TABLE 3:** *W*_*Cog*_ loadings for each measured cognitive variable.

Cognitive variables	*W_Cog_*
DRS	0.26
FAB	0.24
MMSE	0.22
Sequency	0.22
Memory	0.20
Recognition	0.21

The Cognitive latent variable is shown in Table 3 shows the loading of *W*_*Cog*_. The first column is the abbreviation of the cognitive variable and its respective loadings in the second column. Note that the loadings are roughly the same value. Thus, this latent variable is approximately an average of all the tests. The highest loadings are for DRS, followed by FAB, while the minimum loading is for Memory within this narrow range.

The marginal distributions of the loadings for the qEEG latent variables *W*_*qEEG*_ are shown in Figure 2 (frequency) and Figure 3 (space). Figure 2 shows the frequency spread of the latent variable by displaying the minimum and maximum loadings for the 3,244 sources at each of the frequency points. In Figure 2, the *x*-axis is the frequency in Hz with dashed vertical lines for each classical EEG band (delta, theta, low alpha, high alpha, and beta), and the *y*-axis is the standardized latent loadings. The red line shows the highest loadings, and the cyan line shows the minimum loadings for each frequency point. The latent factor maxima and minima are loads distributed over all frequency bands. This pattern suggests that this latent variable is a contrast (weighted linear combination) between different cortical areas at each frequency.

This impression is substantiated by Figure 3 shows the dominant loadings (top 1% positive and negative values) plotted on the cortex and summarized for the classical frequency bands. There is a complex pattern of positive and negative values.

### Mediation Model

We have implemented two mixed effect models with the latent variables for cognition and qEEG (mediator and outcome models).

The mediator model showed that *qEEG* depends positively on dose and education with a *p*-value of 0.003 and 0.02, respectively. In contrast, the fixed effects for age showed a negative coefficient; however, the *p*-value was not significant. Results are given in Table 4. The mediation model’s power analysis showed that the dosage effect is sufficiently powered with 88.00% (95% CI [79.98, 93.64]).

**TABLE 4:** Linear mixed-effects analyses fixed effect estimates for mediation model.

Variables	Estimate	*P*-value
Intercept	–0.128	0.921
Dose	0.496	0.003**
Education	0.066	0.028*
Severity	0.232	0.429
Trial age	–0.023	0.137

Significance codes: 0.001 “**”; 0.01 “*”.

The second model (outcome model) for *Cog* shows a positive dependency on *qEEG* and dose with a *p*-value of <0.0001 and 0.006, respectively (Table 5). The effect of age on Cognition also showed a negative trend; however, the *p*-value was not significant, possibly due to the small sample size and narrow age range. We performed the power analysis for the outcome model as well. The *qEEG* and dose estimate had sufficient power of 100.0% (95% CI [96.38, 100.0]) and 89.00% (95% CI [81.17, 94.38]).

**TABLE 5:** Linear mixed-effects analyses fixed effect estimates for outcome model.

Variables	Value	*P*-value
Intercept	0.150	0.940
*EEG*_1_	3.840	0.000***
Dose	0.987	0.006**
Education	0.067	0.169
Severity	0.670	0.150
Trial age	–0.044	0.083

Significance codes: 0 “***”; 0.001 “**”.

Moreover, we combined these two models using the mediation package. We computed the estimates and quasi-Bayesian confidence intervals for the mediation models (Figure 1), the direct effect pathway (Figure 1: path c), and the mediation effect pathways (Figure 1: paths a and b).

**FIGURE 4:**
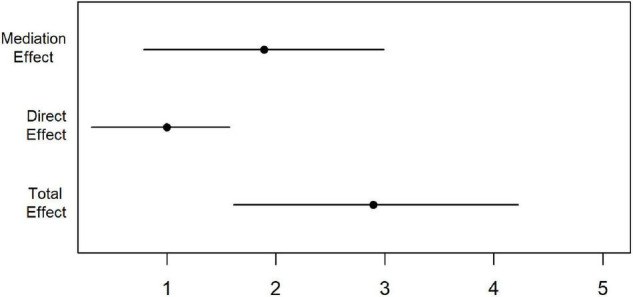
The mediation effect. The indirect path between dose and *Cog via qEEG* shows a more robust and higher estimate than the direct effect. The black diamond symbol shows the point estimate, and the line shows the 95% quasi-Bayesian confidence intervals. The mediation effect explains 66% of the total effect. There is also a significant direct effect between doses and *Cog*, which is not explained by *qEEG*. A positive mediation and direct effect show that a higher dosage is associated with higher latent cognitive scores.

The results showed a positive association between dose and Cognition, meaning a higher value for dose results in higher values for the latent cognitive scores (*Cog*). The beta estimate for the total effect was 2.9 with 95% CI [1.7–4.1] and *p*-value < 0.0001. The 66% of the total effect was mediated by *qEEG*with a *p*-value < 0.0001. The mediation analysis results are summarized in Table 6 and Figure 4. There is also a significant direct effect between dose and *Cog* (*p*-value 0.002), which is not mediated by *qEEG*. Figure 4 shows the point estimates and 95% CI for mediation (indirect), direct, and total effect.

**TABLE 6:** The 95% confidence interval for quasi-Bayesian estimation of the mediation model.

	Estimate	95% CI lower	95% CI upper	*P*-value
Mediation effect	1.899	0.751	3.15	<2e−16***
Direct effect	0.995	0.35	1.6	0.002**
Total effect	2.894	1.63	4.22	<2e−16***
Proportion mediated	0.655	0.404	0.86	<2e−16***

Significance codes: 0 “***”; 0.001 “**”.

## Discussion

This article presents a secondary analysis of a safety trial of the use of Neuroepo in PD patients where the outcomes were cognitive performance and quantitative EEG (qEEG) as a proxy for brain function. Two groups were studied that received a 5 mg dose of Neuroepo (*n* = 15) and the placebo group (*n* = 10). We attempted to answer two main questions: (a) does the administration of Neuroepo have a statistically significant effect on the cognitive outcome?; (b) is this effect, if present, mediated by the qEEG?

Our choice of including qEEG is based on the many studies that report changes in this measure in PD. A causal mediation model is the next logical step, considering the reports that qEEG predicts cognitive worsening (Klassen et al., 2011; Arnaldi et al., 2017; Caviness et al., 2018; Calabrò et al., 2019). Causal analysis is becoming a standard in clinical trial evaluation (VanderWeele, 2015; Goldsmith et al., 2018).

We use a novel approach from multi-omic studies to answer the mediation model questions. Rather than analyzing each measurement domain as an independent module, we used pCCA to uncover latent variables from each domain (Cognition, qEEG) sharing significant association with a correlation of 0.97. This result is not surprising since resting-state EEG has been previously strongly associated with cognitive performance in nondemented PD patients (Zimmermann et al., 2015). This study is relevant to ours as our patients had no cognitive deterioration, the inclusion criteria being an MMSE score >26. This feature distinguishes these studies from others in which progression to dementia was the focus.

From the pattern of the loadings shown in Table 3 and Figures 2–4, the interpretation of the obtained latent variables seems clear. The cognitive latent variable is roughly an average of all cognitive tests. On the other hand, the complex frequency and spatial distribution of the loadings of the qEEG latent variable indicate that no single frequency band or anatomical area is predominant.

The frequency and spatial distribution of the qEEG latent variable are complex, indicating that widespread brain networks must be considered to obtain the optimal correlation with Cognition. The resultant qEEG latent variable is a complex ratio of qEEG source spectral power, a multivariate generalization of the power ratios of isolated frequency bands previously proposed as biomarkers. One example of the use of power ratios is the finding that delta/alpha 3 correlates highly with the MMSE (Babiloni et al., 2017). On the other hand, Gu et al. (2014) tracked differential longitudinal changes between PD patients with MCI and dementia using (Du et al., 2016) alpha/theta. We note that ratios such as these are equivalent to subtractions of log-spectra. Therefore, our latent variable refines these power ratios by constructing an overall contrast of the log spectra over all sources and frequencies. We are studying whether sparse variants of pCCA (Du et al., 2016) can yield a simplified pattern of the qEEG loadings of the latent variable that is more easily interpretable.

In analyzing latent variables and confounders of this 6-months longitudinal study, we note that the PD patients stayed at the same severity stage (I–II of the Hoehn and Yahr scale), maintaining the same medication dose. In any case, age, and severity, were introduced as confounding variables in all linear mixed-effects analyses (LMM). As shown in Table 4, the dose of Neuroepo had a highly significant effect on Cognition. Education had a significant positive coefficient in the LMM, suggesting that it is also a neuroprotective factor. By contrast, the participant’s age entered the trial was negatively correlated to the outcome. Though this result was expected, the corresponding coefficient was not significant, possibly due to the small sample size. We note that the LMM was sufficiently powered, as described in the results.

When broken down by the mediation analysis, we see that the effect of the dose *via* the *qEEG* (as a proxy for brain function) is highly significant and explains 66% of the direct effect. This analysis indicates that some of the effects of Neuroepo dose on Cognition may be due to other factors, which are being explored in future studies.

A possible concern is whether the latent variable analysis might bias the mediation results. We do not consider this case since the pCCA only concentrates the shared variance of Cognition and qEEG and is independent of dose or confounder variables. This result begs the question of studying such a joint latent variate in other populations, normal or other neurodegenerative diseases. This type of latent variable might yield more powerful outcomes for clinical trials.

More extensive clinical studies are needed to fully understand the mechanisms underlying the mediation that qEEG has on the effect of Neuroepo on Cognition. In addition, more preclinical studies on the neuroprotective effects of Neuroepo are required. A similar molecule, EPO, protects nigral dopaminergic neurons (Kanaan et al., 2005; Xue et al., 2009, 2007; Sargin et al., 2010). Neuroepo neuroprotection has also been through mechanisms of action such as the decrease of the inflammatory process, apoptosis cells, oxidative stress, and cell death, achieving a restoration of cerebral homeostasis, which would facilitate an improvement in neural connectivity (Rodríguez Cruz et al., 2010, 2016; Garzón et al., 2018a,b). A focus on effects on qEEG and Cognition would be of great interest.

### Our Study has Several Limitations

First, the clinical trial was designed to test Neuroepo tolerance, where safety and not efficacy were the primary outcome measure. However, phase I studies can have therapeutic potential, mainly in patients and not healthy volunteers (Horton, 2006).

Second, the generalizability of our findings is limited because of the size of the sample. The phase I–II trials use small samples since they are testing novel drugs. However, the output was sufficiently powered. For example, *qEEG*, when analyzed with dose, reached 89% (79.9, 93.6), and analyzed with Cognition reached 100% (96.3, 100).

Third, with such a short treatment and follow-up, we cannot entirely rule out that an underlying idiopathic change in PD progression causes the observed effects on Cognition. The LME contrast would eliminate a factor common to all subjects. This process would have to be distributed differentially in both groups. Larger sample sizes, longer follow-ups, and additional biological measurements would be needed to identify this process.

## Conclusion

In conclusion, in this study, we demonstrated the potential of qEEG for distinguishing the differential effects of pharmacological intervention in PD. To verify these results and delve into the mechanics involved, we launched a phase II-III trial with a larger sample.^2^

## Data Availability Statement

The original contributions presented in the study are publicly available. The EEG data used in this study are stored in BIDS format at OpenNeuro repository: https://openneuro.org/ with the doi: 10.18112/openneuro.ds003194.v1.0.0, for NeuroEPO group and (doi: 10.18112/openneuro.ds003195.v1.0.0) for placebo group. The file with the clinical, demographic and psychological variables is available in the Supplementary Material.

## Ethics Statement

The project had ethics approval from the “Scientific Review Board” at the International Center for Neurological Restoration, from Spanish Centro Internacional de Restauracion Neurologica (CIREN), La Habana, Cuba. The patients/participants provided their written informed consent to participate in this study.

## Author Contributions

IP recruited the patients at CIREN and led the whole clinical trial. LM collected the original EEG recordings at CIREN. TV, PG, and MZ performed the EEG visual inspection and quality control of the EEG datasets for further processing. JB-B, LG, and MZ were responsible EEG data for processing with qEEGt. FR did the new statistical analysis. CL converted the EEG files to BIDS and uploaded them to the OpenNeuro repository. PR and MJ contributed to the supervision of the work and revised the final version of the manuscript. PV-S and MB contributed to the research design and discussion of the analysis strategies and wrote the final version of the manuscript. All authors contributed to the article and approved the submitted version.

## Conflict of Interest

The authors declare that the research was conducted in the absence of any commercial or financial relationships that could be construed as a potential conflict of interest.

## Publisher’s Note

All claims expressed in this article are solely those of the authors and do not necessarily represent those of their affiliated organizations, or those of the publisher, the editors and the reviewers. Any product that may be evaluated in this article, or claim that may be made by its manufacturer, is not guaranteed or endorsed by the publisher.

## Footnotes

1
http://www.neuronicsa.com

2 RPCEC00000233 “Assessing the efficacy and safety of nasal NeuroEpo management in patients disease with Parkinson stage II-III.” https://rpcec.sld.cu/en/trials/RPCEC00000233-En.
